# Sclerostin is an independent risk factor for all-cause mortality in kidney transplant recipients

**DOI:** 10.1007/s10157-020-01956-y

**Published:** 2020-08-20

**Authors:** Shufei Zeng, Torsten Slowinski, Wolfgang Pommer, Ahmed A. Hasan, Mohamed M. S. Gaballa, Yongping Lu, Bernhard K. Krämer, Berthold Hocher

**Affiliations:** 1 Department of Nephrology, Charité-Universitätsmedizin Berlin, Campus Mitte, Berlin, Germany; 2 Fifth Department of Medicine (Nephrology/Endocrinology/Rheumatology), University Medical Centre Mannheim, University of Heidelberg, Heidelberg, Germany; 3 KfH Kuratorium für Dialyse und Nierentransplantation e.V., Bildungszentrum, Neu-Isenburg, Germany; 4 Institute of Nutritional Science, University of Potsdam, Potsdam, Germany; 5 Faculty of Veterinary Medicine, Benha University, Moshtohor, Toukh, Egypt; 6 Department of Nephrology, The First Affiliated Hospital of Jinan University, Jinan University, Guangzhou, China; 7 Institute of Medical Diagnostics, IMD Berlin, Berlin, Germany; 8 Key Laboratory of Study and Discovery of Small Targeted Molecules of Hunan Province, School of Medicine, Hunan Normal University, Changsha, China; 9 Reproductive and Genetic Hospital of CITIC-Xiangya, Changsha, China

**Keywords:** Kidney transplantation, All-cause mortality, Sclerostin

## Abstract

**Background:**

Sclerostin is a hormone contributing to the bone-vascular wall cross talk and has been implicated in cardiovascular events and mortality in patients with chronic kidney disease (CKD). We analyzed the relationship between sclerostin and mortality in renal transplant recipients.

**Methods:**

600 stable renal transplant recipients (367men, 233 women) were followed for all-cause mortality for 3 years. Blood and urine samples for analysis and clinical data were collected at study entry. We performed Kaplan–Meier survival analysis and Cox regression models considering confounding factors such as age, eGFR, cold ischemia time, HbA1c, phosphate, calcium, and albumin. Optimal cut-off values for the Cox regression model were calculated based on ROC analysis.

**Results:**

Sixty-five patients died during the observation period. Nonsurvivors (*n* = 65; sclerostin 57.31 ± 30.28 pmol/L) had higher plasma sclerostin levels than survivors (*n* = 535; sclerostin 47.52 ± 24.87 pmol/L) (*p* = 0.0036). Kaplan–Meier curve showed that baseline plasma sclerostin concentrations were associated with all-cause mortality in stable kidney transplant recipients (*p* = 0.0085, log-rank test). After multiple Cox regression analysis, plasma levels of sclerostin remained an independent predictor of all-cause mortality (hazard ratio, 1.011; 95% CI 1.002–1.020; *p* = 0.0137).

**Conclusions:**

Baseline plasma sclerostin is an independent risk factor for all-cause mortality in patients after kidney transplantation.

**Electronic supplementary material:**

The online version of this article (10.1007/s10157-020-01956-y) contains supplementary material, which is available to authorized users.

## Introduction

Kidney transplant recipients are a subset of patients with chronic kidney disease (CKD). And kidney transplantation is the treatment of choice for end-stage renal disease [1].

Chronic kidney disease-mineral bone disease (CKD-MBD) is associated with increased morbidity and mortality among patients undergoing hemodialysis. Several markers of bone formation and turnover, including fibroblast growth factor 23 (FGF-23), bone-specific alkaline phosphatase and parathyroid hormone (PTH), have been shown to be associated with increased mortality [2–4]. Vascular calcification has been associated with bone and mineral disorders [5].

Sclerostin (SOST) protein is a 213 amino acid protein transcribed from the SOST gene [6, 7]. It is a soluble inhibitor of the Wnt pathway which promotes bone formation. It is among the recently discovered players of the bone-vascular wall cross talk and has gained increased attention lately. Circulating and bone sclerostin mRNA or protein levels in humans and in mice change in response to factors such as hormonal, systemic, and mechanosensory stimuli. Known regulators of sclerostin include PTH, sex steroids, thyroid hormone, corticosteroids, vitamin D, diabetes mellitus, immobilization/spinal cord injury/weight loss/weightlessness, skeletal loading/exercise, malignancies and renal disease [8]. An interesting clinical study showed that serum levels of sclerostin are associated with an increased risk of cardiovascular events and mortality in patients with CKD [9]. However, to the best of our knowledge, the relationship between sclerostin and mortality in renal transplant recipients has never been reported.

Therefore, we analyzed, whether sclerostin is a risk factor of all-cause mortality in kidney transplant recipients. To control for possible confounding, we performed multivariate Cox regression analyses considering known confounding factors [10–14].

## Materials and methods

### Study population

We analyzed a kidney transplant patient cohort as described recently, for details, see [15]. Six hundred deceased donor kidney transplant recipients (367 men, 233 women) were enrolled in a prospective study, which was conducted at the transplant clinic Charité-Mitte, Berlin, Germany, between August and December 2012. The patients gave their informed consent. The study protocol was approved by the local ethical committees (approval number 2012-327). Patients were excluded if they had any acute infection, malignancy, acute rejection (patients who have biopsy-proven acute rejection within 6 months after transplant), acute myocardial infarction, pulmonary edema, heart failure at the time of blood sampling. At the beginning of the study, blood was collected and stored at − 20 °C until use. The patients were followed for all-cause mortality for 3 years.

### Data sources and measurement

Demographic data for recipients and donors (cold ischemia time, HLA mismatches, donor's age, panel reactive antibodies, recipient's age and sex, and transplant survival, underlying kidney disease etc.) were extracted from hospital records and the Euro-transplant records of the patients. Albumin, creatinine, cholesterol, HbA1c, 1,25(OH)2D, calcium, phosphate, iPTH, fasting blood glucose and urinary protein were measured by standardized methods in the central clinical laboratories of Charité. All the used assays were subject to regular quality control. Sclerostin concentration was analyzed with the use of a commercial ELISA (BI-20492, Biomedica Medizinprodukte GmbH, Vienna, Austria), according to the manufacturer’s instructions.

### Statistical analysis

We analyzed blood levels of calcium, phosphate, HbA1c, eGFR, albumin, patient age and cold ischemia time using the area under the receiver operating characteristic (ROC) curve to obtain optimal cut-off values for these parameters [16]. When the ROC curve is plotted with 1—specificity on the abscissa and the corresponding values for sensitivity on the ordinate, the point of the ROC curve closest to the upper left corner of the coordinate system (where sensitivity and specificity equal 1) represents the best cutoff value. These cutoff values were calculated and used to transform individual continuous parameters into binary endpoints.

Plasma levels of sclerostin in survivors and nonsurvivors were compared using independent *T* test. Survival rates were tested for significant differences with the log-rank test using the Kaplan–Meier method. Univariate Cox proportional hazards modeling was performed to investigate the relationship between clinical measurements and mortality based on previously identified researches [10–14]. To control for possible confounding, we first performed multivariate Cox regression analyses considering age, eGFR, and plasma albumin. Based on these confounding factors, we add plasma calcium and phosphate in model B to test the stability of the independent relationship between sclerostin and all-cause mortality. Furthermore, we considered all parameters that were significant predictors in univariate analysis (age, eGFR, plasma albumin, phosphate, calcium, HbA1c, and cold ischemia time) as confounding factors in model C. In subgroup analysis, we grouped according to the cutoff value of sex, eGFR, phosphate and calcium respectively and performed multivariate Cox regression analysis of model C in each subgroup.

### Results

A total of 600 stable renal transplant recipients, 367 males and 233 females, aged 20–87 years, were included in this study. Patient demographics and relevant clinical and biochemical parameters are summarized in Table 1. At study entry, mean age of patients was 54.57 ± 14.54 years old, and mean estimated glomerular filtration rate (eGFR) was 44.41 ± 18.03 mL/min/1.73 m^2^. Meantime since transplantation was 89.85 ± 75.32 months, and time on dialysis before renal transplantation was 49.51 ± 35.23 months. Mean plasma sclerostin concentration was 48.58 ± 25.67 pmol/L. The concentration of sclerostin was significantly (*p* = 0.0003) higher in male (51.58 ± 25.16 pmol/L) compared to female (43.85 ± 25.79 pmol/L) patients. Sclerostin concentrations were slightly higher in patients treated with Cyclosporin A versus Tacrolimus treated patients (see supplementary Fig. 1). Sixty-five patients died during the observation period.

**Table 1:** Clinical data and plasma parameters in all 600 renal transplant recipients at study entry

	Mean (SD) or *n*	Median (interquartile range)
Age at study entry (years)	54.57 ± 14.54	55.00 (22)
Sex male/female (*n*)	367/233	367/233
Donor age (years)	49.37 ± 15.82	52.00 (23)
Time on dialysis (month)	49.51 ± 35.23	48.00 (59)
Time post-transplantation (month)	89.85 ± 75.32	60.00 (77)
Cold ischemia time (h)	10.06 ± 7.34	9.78 (8.88)
eGFR (mL/min/1.73 m^2^)	44.41 ± 18.03	43.00 (26)
Plasma albumin (g/dL)	4.55 ± 0.35	4.50 (0.50)
Plasma creatinine (mg/dL)	1.75 ± 0.72	1.57 (0.75)
Total cholesterol (mg/dL)	223.12 ± 52.96	220.00 (77)
HbA1c (%)	5.89 ± 0.85	5.80 (0.80)
Plasma 1,25(OH)_2_D (pmol/L)	96.17 ± 54.39	90.00 (72.50)
Plasma calcium (mmol/L)	2.48 ± 0.19	2.46 (0.22)
Plasma phosphate (mmol/L)	0.88 ± 0.26	0.84 (0.31)
Plasma iPTH (pg/mL)	113.85 ± 129.49	85.31 (93.12)
Plasma sclerostin (pmol/L)	48.58 ± 25.67	45.90 (25.75)
Fasting blood glucose (mg/dL)	95.61 ± 35.10	88.00 (32)
Urinary protein (mg/24 h)	366.34 ± 646.56	167.00 (212)

Values are presented as mean (SD) and median (interquartile range) or *n*

eGFR, estimated glomerular filtration rate; HbA1c, hemoglobin A1c; iPTH, intact parathyroid hormone

The underlying renal diseases are as follows: 474 of the 600 patients had primary renal diseases. Fifty-eight patients had secondary renal disease. In 68 patients, the underlying renal disease was unknown. More than 60 percent of patients had the primary glomerular disease, polycystic kidney disease and interstitial kidney disease. The underlying kidney diseases are described in Supplementary Table 1.

**Fig. 1:**
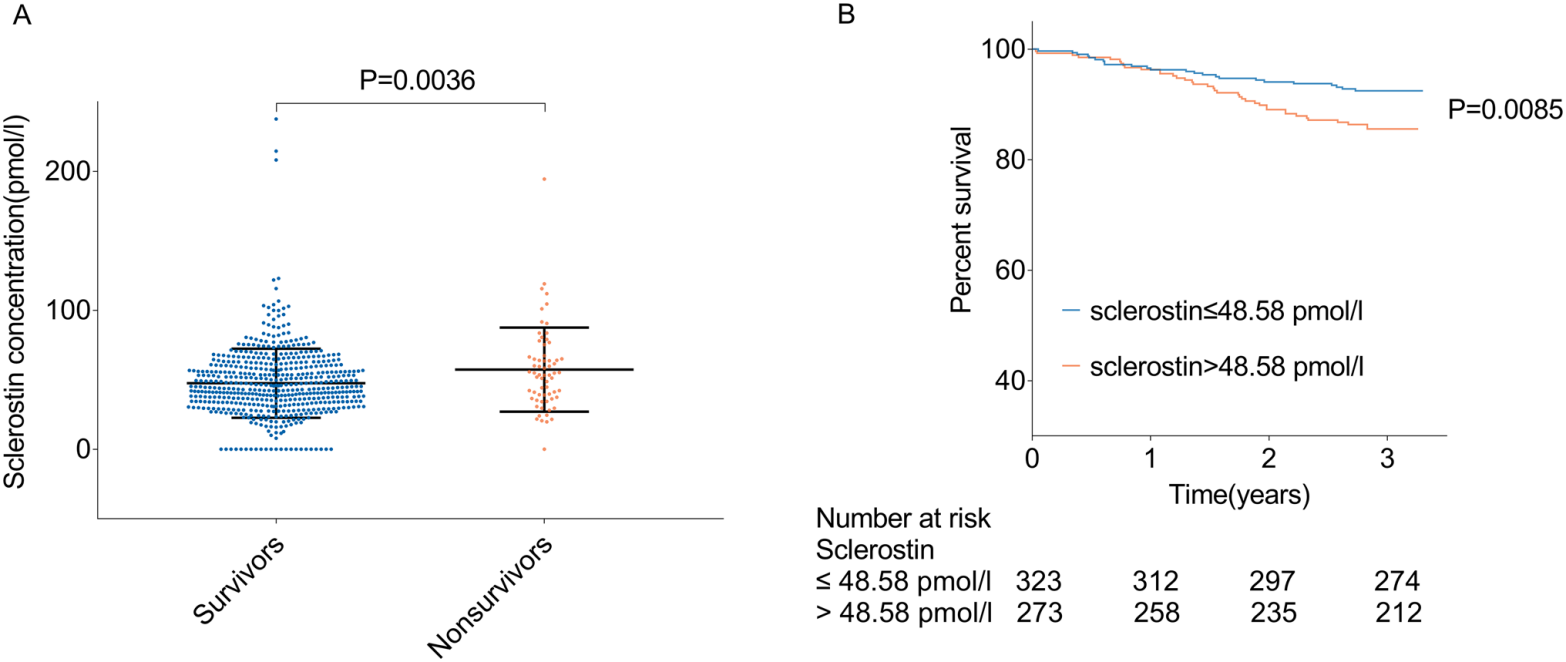
Differences in plasma sclerostin levels between survivors and non-survivors and survival rates in patients with higher and lower levels of sclerostin. **a** Individual sclerostin values in patients who died and survived during the follow-up period. **b** Kaplan–Meier curves, according to mean plasma sclerostin concentration (48.58 pmol/L), for all-cause mortality. Blue lines, patients with plasma sclerostin levels of ≤ 48.58 pmol/L; orange lines, patients with plasma sclerostin levels of > 48.58 pmol/L. Survival rates were compared with the log-rank test

Nonsurvivors had higher plasma levels of sclerostin than survivors (Fig. 1; *p* = 0.0036). Sclerostin concentrations above 48.58 pmol/L were significantly associated with all-cause mortality in renal transplant patients (Fig. 1; *p* = 0.0085, log-rank test).

We built three multivariate Cox regression models by gradually adding confounding factors, which were parameters that were statistically significant predictors in the univariate analysis (Table 2). These three multivariate Cox regression models showed that sclerostin concentrations were independently associated with all-cause mortality in stable renal transplant recipients (Table 3; model A: hazard ratio, 1.013; 95% CI 1.004–1.021; *p* = 0.0052; model B: hazard ratio, 1.011; 95% CI 1.002–1.020; *p* = 0.0136; model C: hazard ratio, 1.011; 95% CI 1.002–1.020; *p* = 0.0137).

**Table 2:** Univariate Cox regression analysis of risk factors for all-cause mortality in kidney transplant recipients

	HR	95% CI	*p*
Age	1.074	1.049–1.098	< 0.0001
Sex	1.608	0.929–2.783	0.0896
Cold ischemia time	1.052	1.021–1.083	0.0008
Donor age	1.017	1.000–1.035	0.0506
Time on dialysis	1.005	0.998–1.012	0.1710
Time after transplantation	1.002	0.999–1.005	0.1528
Total cholesterol	0.997	0.992–1.002	0.2861
eGFR	0.974	0.958–0.990	0.0016
Urine protein	1.000	0.999–1.000	0.6089
Albumin	0.340	0.133–0.873	0.0249
HbA1c	1.458	1.166–1.823	0.0009
1,25(OH)_2_D	1.001	0.996–1.006	0.6877
Calcium	0.264	0.078–0.896	0.0327
Phosphate	3.973	1.761–8.963	0.0009
iPTH	1.000	0.998–1.002	0.7863
Sclerostin	1.012	1.005–1.018	0.0004

Patients were followed for all-cause mortality for 3 years

eGFR, estimated glomerular filtration rate; HbA1c, hemoglobin A1c; iPTH, intact parathyroid hormone

**Table 3:** Cox proportional hazards analysis of the relevant factors with all-cause mortality in renal transplant recipients

	HR	95% CI	*p*
*Model A*			
Age (> 59.5 years)	0.271	0.121–0.610	0.0016
eGFR (> 49.5 mL/min/1.73 m^2^)	0.732	0.320–1.674	0.4595
Albumin (> 4.45 g/dL)	2.760	1.287–5.921	0.0091
Sclerostin (pmol/L)	1.013	1.004–1.021	0.0052
*Model B*			
Age (> 59.5 years)	0.270	0.121–.0603	0.0014
eGFR (> 49.5 mL/min/1.73 m^2^)	0.695	0.304–1.589	0.3885
Albumin (> 4.45 g/dL)	2.670	1.220–5.842	0.0140
Phosphate (> 0.845 mmol/L)	0.816	0.396–1.681	0.5811
Calcium (> 2.435 mmol/L)	1.660	0.789–3.494	0.1819
Sclerostin (pmol/L)	1.011	1.002–1.020	0.0136
*Model C*			
Age (> 59.5 years)	0.262	0.112–0.611	0.0019
eGFR (> 49.5 mL/min/1.73 m2)	0.645	0.277–1.507	0.3115
Albumin (> 4.45 g/dL)	2.756	1.245–6.097	0.0124
Phosphate (> 0.845 mmol/L)	0.766	0.367–1.602	0.4793
Calcium (> 2.435 mmol/L)	1.651	0.774–3.522	0.1945
Cold ischemia time (> 11.505 h)	1.082	0.515–2.273	0.8344
HbA1c (> 6.05%)	0.965	0.457–2.039	0.9257
Sclerostin (pmol/L)	1.011	1.002–1.020	0.0137

Multiple proportional hazards regression analyses (Cox regression; enter). Patients were followed for all−cause mortality for 3 years. Models A, B and C show that sclerostin levels are independently associated with all-cause mortality after adjustment for confounding variables

Since some studies [17–19] reported that sclerostin concentrations in male CKD patients are higher than in female CKD patients, we performed an additional multivariate Cox regression model including additional sex as a confounding factor. This model likewise confirmed that sclerostin is an all-cause mortality risk factor after kidney transplantation (see supplementary Table 2). We also build another Cox regression model by adding time on dialysis and time after transplantation to model C, sclerostin remain significant as an independent risk factor for all-cause mortality (see supplementary Table 3).

**Fig. 2:**
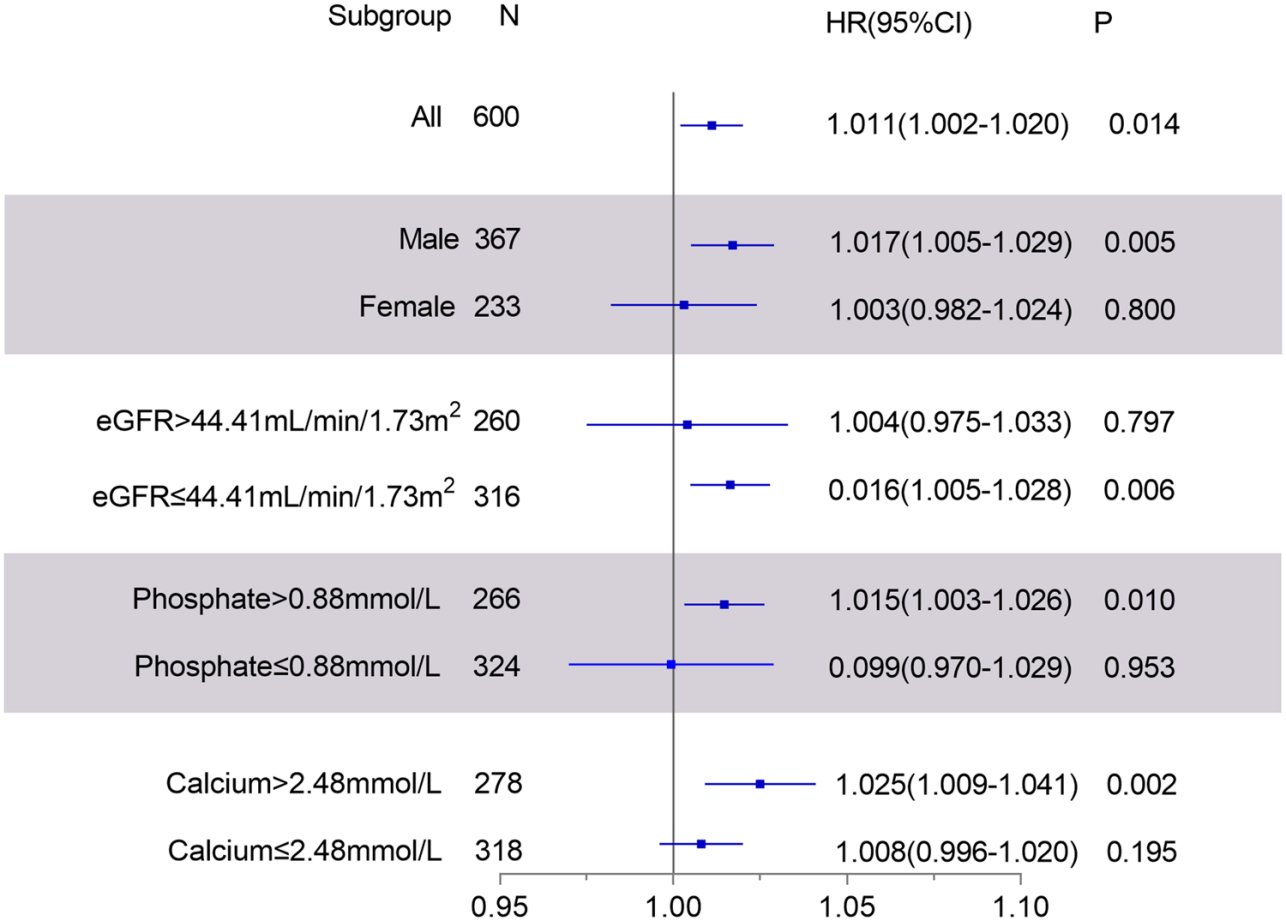
Association between sclerostin and all-cause mortality among subgroups. HRs for all-cause mortality by sex, eGFR, phosphate and calcium. Adjusted for age, eGFR, plasma albumin, phosphate, calcium, HbA1c, and cold ischemia time

Subgroup analysis (Fig. 2) revealed that sclerostin is, in particular, an important all-cause mortality risk factor among male patients, among patients with lower GFR, among those with higher calcium, and among those with higher phosphate at study entry.

### Discussion

This study demonstrated that elevated baseline plasma sclerostin is an independent risk factor of all-cause mortality in patients after kidney transplantation.

Patient demographics and relevant clinical and biochemical parameters indicate that our study population was representative for a typical European post-transplantation cohort. The findings of our study are thus of general applicability [20, 21].

In the non-transplanted CKD population, conflicting results concerning sclerostin-associated mortality risk were described [22–25]. Our study showed that elevated baseline plasma sclerostin is an independent risk factor of all-cause mortality in patients after kidney transplantation. There are at least two main reasons potentially explaining the differences: Most of these studies [22–25] are substantially smaller than ours make any result subject to potential chance finding. In addition, the investigated populations were different to our cohort: CKD patients on hemodialysis or peritoneal dialysis, and non-dialysis CKD patients.

When analyzing male and female patients separately (Fig. 2), we found that sclerostin is associated with all-cause mortality just in male patients. This might be at least partially due to the fact that sclerostin concentrations are higher in male than female transplant recipients. Higher blood sclerostin concentrations in males were also reported in non-transplanted CKD patient cohorts [17–19]. Females might have lower sclerostin concentrations due to the estradiol mediated decreases in sclerostin seen in humans [26]. Sex differences with regard to all-cause mortality have also been described for the hormone relaxin in patients on hemodialysis [27].

Several other studies in dialysis cohorts analyzed the association of sclerostin with all-cause and cardiovascular mortality. These studies [23–25, 28–33], however, lead to conflicting data. So far, nine studies have analyzed the relationship between sclerostin and all-cause mortality in dialysis patients, five showed that sclerostin is associated with all-cause mortality [23–25, 32, 33], and four of them show no association [28–31]. Four of these studies also analyzed the relationship between sclerostin and cardiovascular mortality, three studies saw an association of sclerostin with cardiovascular mortality [23, 25, 28], but one study did not find this association [29]. Our study is the first to study the association of sclerostin with all-cause mortality in transplanted patients and clearly showed that sclerostin is an independent all-cause mortality risk factor in this population.

Fifty to 60 percent of deaths among kidney transplant recipients are directly attributable to cardiovascular disease, which has a reported incidence of approximately 1 per 100 person-years at risk [34, 35]. The development of vascular calcification and arterial stiffness leads to an increased incidence of cardiovascular disease. Sclerostin is a soluble inhibitor of Wnt signaling pathway and Wnt signaling pathway is thought to be involved in the development of vascular calcification and cardiovascular disease [36].

It was suggested that sclerostin—as an inhibitor of Wnt signaling pathways might play a role in the development of aortic valve and vascular calcifications. A study conducted by Hampson et al. [37] supports this hypothesis. In adjusted linear regression analysis, sclerostin was positively associated with abdominal aortic calcification (AAC). Furthermore, Kuipers et al. [38] showed that increases by one standard deviation of sclerostin levels are associated with  1.61 times greater risk of coronary artery calcification. Sclerostin is an inhibitor of the LRP5/6 coreceptors and the intracellular part of LRP5/6 contains multiple phosphorylation sites, the phosphorylation of which is a key step in the initiation of the signal transduction via WNT/β-catenin signalling [39]. LRP6 was also found to limit the process of vascular calcification. Indeed, the conditional deletion of LRP6 in the vascular smooth muscle cell lineage increased aortic calcium content in diabetic LDLR−/− mice [40].

Our study demonstrated that sclerostin is an independent risk factor of all-cause mortality in kidney transplant recipients. Preclinical studies—see above—suggest that an inhibition of the transmission of the Wnt signaling pathway by inhibiting LRP5/6 co-receptors, resulting in increased vascular calcification and cardiovascular events. Whether this pathway might explain our findings needs to be explored in more detail.

A humanize monoclonal antibody targeting sclerostin is currently tested in clinical trials, Blosozumab (ClinicalTrials.gov Identifier: NCT01144377). It has been studied with regards to the treatment of osteoporosis in postmenopausal women with low bone density [41]. In addition, very recent research shows that SOST-Fc vaccination might be a therapeutic approach for preventing osteoporosis [42]. So far, it has not been established how anti-sclerostin antibodies affect vascular calcifications [43]. These studies will provide first clinical information’s how sclerostin blocking antibodies might affect vascular calcification or vascular stiffness.

Several limitations should be taken into consideration when the results of this study are interpreted. Although we analyzed 600 patients, the sample size may be a limitation in our study, which might affect the results of the Cox regression analysis of multiple risk factors. We only have valid data on all-cause mortality. Data on specific reasons of death, such as cardiovascular mortality and cardiovascular events, however, were not available. Moreover, our study was a single-center study, thus center-specific effects could not be excluded. Thus, confirmation in an independent cohort is important.

Taken together, our study is the first to investigate the potential role of sclerostin in patients after kidney transplantation. Our data suggest that elevated baseline plasma sclerostin is an independent risk factor for all-cause mortality in patients after kidney transplantation. This is of particular clinical impact, since anti-sclerostin antibodies are currently under clinical investigation.

## Electronic supplementary material

Below is the link to the electronic supplementary material.

Supplementary file1 (DOCX 72 kb)
