# Supplementary material for: Sclerostin is an independent risk factor for all-cause mortality in kidney transplant recipients
Source: Clin Exp Nephrol. 2020 Aug 20;24(12):1177–83. doi: 10.1007/s10157-020-01956-y (PMC7599189; doi:10.1007/s10157-020-01956-y)
Supplement: Supplementary file 1 — Supplementary file1 (DOCX 72 kb) [file 10157_2020_1956_MOESM1_ESM.docx]

**Supplementary Table 1. The underlying renal diseases in all 600 renal transplant recipients.**

|  | Cases |
| --- | --- |
| **primary renal disease** | **474** |
| Primary glomerular disease | 253 |
| polycystic kidney disease | 77 |
| interstitial kidney disease | 77 |
| hereditary nephritis | 16 |
| reflux nephropathy | 15 |
| analgesic nephropathy | 8 |
| Hypoplasia | 8 |
| hemolytic uremic syndrome | 7 |
| Hydronephrosis | 3 |
| Nephrocalcinosis | 3 |
| Cystinosis | 2 |
| urethral valves | 1 |
| renal amyloidosis | 1 |
| Nephrolithiasis | 1 |
| Oxalosis | 1 |
| Nephronophthisis | 1 |
| **secondary renal disease** | **58** |
| hypertensive nephrosclerosis | 20 |
| [diabetic nephropathy](http://pmmp.cnki.net/Resources/CDDPdf/dis%5Cbase%5C%E5%86%85%E5%88%86%E6%B3%8C%E7%A7%91%5C%E7%B3%96%E5%B0%BF%E7%97%85%E6%80%A7%E8%82%BE%E7%97%85.pdf) | 18 |
| systemic lupus erythematosus (SLE) nephropathy | 8 |
| anaphylactoid purpura nephritis | 7 |
| [hyperuricemic nephropathy](http://pmmp.cnki.net/Resources/CDDPdf/dis%5Cbase%5C%E8%82%BE%E5%86%85%E7%A7%91%5C%E9%AB%98%E5%B0%BF%E9%85%B8%E8%A1%80%E7%97%87%E8%82%BE%E7%97%85.pdf) | 2 |
| kidney tumor | 2 |
| renal involvement in scleroderma | 1 |
| **Unknown** | **68** |

^Values are presented as n.^

**Supplementary Table 2. Cox proportional hazards analysis of the relevant factors with all-cause mortality in renal transplant recipients.**

|  | HR | 95%CI | P |
| --- | --- | --- | --- |
| Age (>59.5y) | 0.251 | 0.107-0.588 | 0.001 |
| Sex (male versus female) | 2.087 | 0.921-4.729 | 0.078 |
| eGFR (>49.5mL/min/1.73 m2) | 0.637 | 0.269-1.505 | 0.304 |
| Albumin (>4.45g/dl) | 2.734 | 1.243-6.014 | 0.012 |
| Phosphorus（>0.845mmol/L） | 0.719 | 0.346-1.495 | 0.377 |
| Calcium (>2.435 mmol/L) | 1.749 | 0.814-3.758 | 0.152 |
| Cold ischemia time (>11.505h) | 0.998 | 0.473-2.107 | 0.996 |
| HbA1c (>6.05%) | 0.976 | 0.465-2.05 | 0.949 |
| Sclerostin (pmol/l) | 1.011 | 1.002-1.021 | 0.021 |

**Supplementary Table 3. Cox proportional hazards analysis of the relevant factors with all-cause mortality in renal transplant recipients.**

|  | HR | 95%CI | P |
| --- | --- | --- | --- |
| Age (>59.5y) | 0.169 | 0.062-0.463 | 0.001 |
| eGFR (>49.5mL/min/1.73 m2) | 0.475 | 0.178-1.269 | 0.137 |
| Albumin (>4.45g/dl) | 3.801 | 1.533-9.427 | 0.004 |
| Phosphorus（>0.845mmol/L） | 0.62 | 0.269-1.427 | 0.261 |
| Calcium (>2.435 mmol/L) | 1.224 | 0.533-2.814 | 0.633 |
| Cold ischemia time (>11.505h) | 1.712 | 0.722-4.056 | 0.222 |
| HbA1c (>6.05%) | 1.412 | 0.603-3.306 | 0.427 |
| Time on dialysis(>58.5months) | 0.587 | 0.256-1.344 | 0.207 |
| Time after transplantation(>94.5months) | 0.880 | 0.350-2.211 | 0.785 |
| Sclerostin (pmol/l) | 1.014 | 1.005-1.024 | 0.004 |


**Supplementary Figure 1. Plasma sclerostin concentration in patients using different immunosuppressive drugs.**
